# Notes and Notices

**Published:** 1894-02

**Authors:** 


### NOTES AND NOTICES.

STEARNS' CALENDAR FOR 1894.—The Christmas greeting which Frederick Stearns & Co., of Detroit, Mich., are sending to their customers, is of a character which far exceeds the many elegant expressions of good wishes which this firm sent out in former years, and is of exceptional interest on account of the introduction of a new feature in pictorial art, one to which much scientific research and effort have been directed during late years—that of photography in original colors. Stearns' calendar for 1894 is, we believe, the first example of the application of this new process which has been offered the public, and as such it possesses an interest aside from its artistic value. As regards the latter, however, a happy selection has been made in the reproduction of "The Rivals," by F. P. Michetti, a subject in which is included the inimitable coloring which nature gives to a pleasing landscape with that of two gayly costumed peasant girls whose graceful attitudes suggest the title of the picture. The details which attend the process are referred to at length in a small folder which accompanies the calendar. With the exception of the making of the plates, all the work upon the calendar was done in the press room and bindery of the firm, and the result is certainly one in which they can take considerable pride. All regular customers of the firm will receive a copy of this elegant calendar. The feature of expense in the production of such work being a considerable one, the supply is, therefore, necessarily limited, and duplicates can only be furnished upon receipt of 25 cents to cover actual cost of production, postage, and packing.—*Pharm. Era.*

REMEDIES HAVING COLD SALIVA.

December 12th, 1893.

EDITOR OF THE HOMŒOPATHIC PHYSICIAN:

In the proceedings of The New York Homœopathic Union reported in THE HOMŒOPATHIC PHYSICIAN for December, 1893, pages 594 to 598, you ask what remedy has *cold saliva.* In my private repertory I find *cold saliva flows from mouth: Phyto., Calad.* In Lippe I find, *cold saliva: Asar., Cist.* I do not find in Hering's Con., nor in Cowperthwait's Mat. Meds. anything about either. These unusual symptoms should be better known, as they very frequently will decide in the choice of a remedy. Such things always find a place in my repertory, and the time thus given saves me both much time and labor in searching for such symptoms. A repertory article of such unusual symptoms would do much good. Fraternally,

W. A. YINGLING.

